# Reliability and validity of the Londrina activities of daily living protocol in individuals with systemic sclerosis

**DOI:** 10.1007/s10067-025-07309-y

**Published:** 2025-01-09

**Authors:** Yasemin Gedikli, Buse Ozcan Kahraman, Merih Birlik, Serap Acar, Sema Savci

**Affiliations:** 1 Faculty of Physical Therapy and Rehabilitation, Department of Physiotherapy and Rehabilitation, Dokuz Eylül University, Izmir, Turkey; 2 Faculty of Health Sciences, Department of Physiotherapy and Rehabilitation, Selcuk University, Konya, Turkey; 3 Faculty of Physical Therapy and Rehabilitation, Cardiopulmonary Department, Dokuz Eylül University, Izmir, Turkey; 4 Department of Internal Disease, Division of Immunology and Rheumatology, Faculty of Medicine, Dokuz Eylül University, Izmir, Turkey; 5 Faculty of Health Sciences, Department of Physiotherapy and Rehabilitation, Acıbadem Mehmet Ali Aydınlar University, Istanbul, Turkey

**Keywords:** Systemic sclerosis, activities of daily living, functional capacity, upper extremity muscle strength

## Abstract

**Purpose:**

To investigate the validity and reliability of the Londrina ADL Protocol in patients with systemic sclerosis (SSc).

**Methods:**

The study included 39 individuals with SSc and 30 healthy participants aged 18-70 years. Performance-related ADL assessment was performed with the Londrina ADL Protocol which was performed twice by the same rater and energy expenditure during the test with the Dynaport Move Monitor device. Functional capacity (6 Minute Walk Test-6MWT), upper extremity muscle strength (hand-held dynamometer), hand and finger grip strength (hand dynamometer and pinch meter), upper extremity functionality (Q-Dash Questionnaire and 9 Hole Peg Test), self-reported ADL (Milliken ADL Scale (MAS)) and quality of life (SF-36 questionnaire) were evaluated.

**Results:**

Upper extremity muscle strength, functional capacity, upper extremity functionality, and quality of life of individuals with SSc were found to be significantly lower and the time spent by individuals with SSc in the Londrina ADL Protocol was lower and the time spent by individuals with SSc in the Londrina ADL Protocol was significantly longer compared to the healthy group (*p*<0.05). Performance in the the Londrina ADL had high intraclass correlation coefficient ICC=0.813. A moderate negative correlation was found between the time to complete the Londrina Protocol and 6MWT (*p*<0.05), and a weak negative correlation was found between dominant side hand grip strength (*p*<0.05) and MAS (*p*<0.05) in individuals with SSc. A moderate positive correlation was found between the Londrina Protocol and the 9-Hole Peg Test for the dominant side (*p*<0.05).

**Conclusion:**

The Londrina Protocol is a valid and reliable method, hence, this makes it appropriate for an objective, performance-based evaluation of ADL in SSc population.

**Table:** 

**Key Points** • *The results obtained by reapplication the Londrina ADL Protocol under the same conditions showed that the Londrina ADL Protocol is a highly reliable assessment method in scleroderma patients.* • *Considering the activities and protocol content of the Londrina ADL Protocol, it is thought to show functional performance better than the Glittre ADL test and 6MWT.* • *The Londrina ADL Protocol is appropriate for objective and performance-based assessment of ADL in the SSc population for both research and clinical practice.*

## Introduction

Systemic sclerosis (SSc) is a connective tissue disease characterized by fibrosis of the skin, musculoskeletal system, and internal organs associated with vasculopathy and late-stage tissue atrophy [1]. It is classified into two main subgroups: limited cutaneous scleroderma and diffuse cutaneous scleroderma, which are widely accepted. In limited SSc, fibrosis is limited to the hands, arms, and face. Diffuse SSc is a rapidly progressive form that affects one or more internal organs and has a wide spread in the skin [2]. Cardiac involvement may lead to arrhythmia, congestive heart failure, and pericarditis, while pulmonary involvement may lead to pulmonary arterial hypertension, interstitial lung disease, decreased forced vital capacity, and pulmonary carbon monoxide diffusion capacity [3]. Contractures and deformities of the hand are the most common musculoskeletal disorder, and impaired hand function, characterized by decreased range of motion, dexterity, and grip strength has been identified by patients with SSc as the main source of difficulty in activities of daily living [4]. When musculoskeletal involvement accompanies pulmonary and cardiac involvement as a result of systemic involvement, the functionality of individuals with SSc is restricted and this leads to impaired activities of daily living (ADL) and physical inactivity [5]. This recurrent process between a sedentary life and the nature of scleroderma disease becomes a vicious circle and limits functional exercise capacity in this patient group. One of the main manifestations of SSc is contractures and deformities of the hands, feet, and joints and facial disfigurement. These represent a considerable burden for patients’ disability status, as they cause major impairment in daily functioning and activities [6–9]. Painful digital ulcers, flexion contractures of the fingers, reduced joint movement and mouth opening can limit daily activities, making even the simplest daily actions difficult (dressing, showering, eating, talking, etc.) [10]. ADL can primarily be assessed using performance-based tests, where an individual’s performance is evaluated while they engage in standardized activities, and questionnaires that rely on self-reported responses to measure limitations in ADL. Performance-based tests provide the advantage of observing actual performance and identifying potential limitations during these activities. One of the methods used to assess ADL, the Glittre ADL Test (GAT), is more similar to the 6MWT, possibly due to the test design encouraging individuals to walk as fast as possible, whereas the Londrina ADL Protocol includes more upper extremity activity than the GAT and the 6MWT. Considering that the 6MWT represents functional exercise capacity and the Londrina ADL Protocol represents functional performance, the Londrina ADL Protocol represents the individual’s real life and performance of daily ADL activities [11]. This measurement method, which consists of the most frequently used activities in daily life, assesses ADL performance objectively and reliably. Therefore, our aim was to demonstrate ADL impairment in these individuals using a performance-related test with objective data and to investigate the reliability and validity of the Londrina ADL Test in individuals with SSc.

## Methods

### Participants

Thirty-nine patients with SSc diagnosed according to 2013 ACR/EULAR classification criteria from the Department of Rheumatology, Dokuz Eylül University, and 30 healthy individuals were recruited to this study from July 2023 to April 2024. Forty-six patients with systemic sclerosis were screened. Five patients were excluded because they did not fulfil the inclusion criteria (2 patients were older than 70 years, 3 patients had an additional diagnosis of rheumatoid arthritis) and 2 patients did not want to participate in the study. For the control group, 40 participants were screened. Ten people were excluded due to systemic-chronic diseases.A G-Power analysis was used to determine the sample size. For 80% power, a margin of error of 0.05 and an effect size of 0.76, the minimum number of patients required for both groups was calculated as 26. The inclusion criteria included being between 18 and 70 years of age, being clinically stable and having the ability to adapt to the tests (visual, cognitive, cooperative). Patients with cognitive and psychiatric problems that may affect the study, neurologic and/or musculoskeletal problems that may affect the study, previous upper extremity surgery, known additional rheumatologic diseases, contractures severe enough to prevent grasping and pregnant women were excluded. Healthy participants were voluntarily selected from the relatives of patients and hospital staff who met the inclusion criteria. Purposive sampling method was applied because individuals who met certain inclusion criteria were included in both groups. Non-Interventional Research Ethics Committee of Dokuz Eylül University approved the study (Approval Date: 05.07.2023 and Approval Number: 2023/22–04). Informed consent was obtained from all the participants before entering the study.

### Disease severity assessment

Medsger Systemic Sclerosis Severity Scale (MSS) was used to assess disease activity. The scale consists of a total of 9 parameters and each parameter is scored between 0 and 4. When a score of 3 or above is obtained, the disease is considered to be severe [12].

### Activities of daily living assessment

Activities of daily living (ADLs) were assessed both performance-based and patient-reported. For performance-based assessment of ADL, the Londrina ADL Protocol was used. It consists of different activities including upper and lower extremity and trunk stretching-bending-rotation movements. The protocol is composed of 5 activities, including walking with and without the addition of extra weight to the body, moving objects on shelves and on a table, and hanging clothes on a clothesline.

Individuals were asked to perform the tasks in the protocol in the same way as they do at home and the completion times were recorded both for the total time and for each station separately. Heart rate, blood pressure, scores for the sensation of dyspnoea and leg fatigue (modified Borg scale 0–10) were measured before and immediately after each test. Energy expenditure during the protocol was recorded with the DynaPort Move Monitor (McRoberts, The Hague, the Netherlands) device [11]. For energy expenditure, basal metabolic rate, diet induced thermo genesis, activity-related energy expenditure, total energy expenditure were measured. The Londrina ADL test was performed once in the healthy group and twice in the patients with scleroderma, one hour apart.

Questionnaire-based activities of daily living (ADLs) of the individuals were assessed using the MAS. The scale includes 47 items and ability and necessity are considered separately for each item. Scoring is based on 5 points for ability level and 3 points for necessity level. The maximum total score is 705. The Turkish version of this questionnaire was validated by Akel et al. [13].

### Functional capacity assessment

Functional capacity was assessed with the 6MWT, performed as recommended by the American Rheumatology Association. Individuals were asked to walk for six minutes in a 30-m-long corridor at the fastest speed they could walk. The equations formulated by Trooster et al. were used to calculate the expected walking distance of the individuals [14]. All tests were performed on the same day. The next test was performed after the participant’s vital signs returned to baseline (approximately 1 h).

### Upper extremity muscle strength assessment

Muscle strength was measured for shoulder flexor, shoulder abductor, hand and finger grip muscles. Jamar hand dynamometer (Patterson Medical, Warrenville, USA) was used to measure hand grip strength and Jamar finger pinchmeter (Baseline, USA) was used to measure finger grip strength. The measurements were made as recommended by the American Association of Hand Therapists (AETD) and the average of 3 measurements was taken and recorded in kg [15].

Shoulder flexor and abductor muscle strength was measured with the MicroFet (Hoggran, USA) while sitting with the shoulder flexed 90° and the elbow fully flexed with the shoulder abducted approximately 80°, respectively [16].

### Assessment of upper extremity functionality

Questionnaire-based assessment of upper limb functionality was performed with the Quick-DASH. Scoring was done between 1 and 5. A score of 1 indicates no difficulty during the activity, whereas a score of 5 indicates inability to perform the activity at all [17].

Performance-based assessment of upper extremity functionality was evaluated with the 9-Hole Peg Test. The participant was asked to pick up the sticks in a separate compartment, place them one by one into the holes in the square block as quickly as possible and pick them back up one by one. The elapsed time was measured with a stopwatch and recorded [18].

### Assessment of quality of life

Short Form-36 (SF-36) questionnaire was used for quality of life assessment. This questionnaire consists of a total of 36 questions divided into 8 sub-parameters. The total score in the questionnaire ranges from 0 to 100 and an increase in score represents good health status [19].

### Statistical analysis

The Shapiro–Wilk test and histogram graphics were used to check the normality of the data. The demographic and clinical characteristics of the participants were reported using descriptive statistics. The intraclass correlation coefficient (ICC) with two-way random effects and absolute agreement methods was used to assess the testretest reliability [20]. Ninety-five per cent Confidence Interval (95% CI) was calculated to investigate the measurement variability. Differences between the systemic sclerosis and healthy groups were tested by independent sample *t*-test (parametric conditions) or Mann–Whitney *U* test (non-parametric conditions), and correlations between activities of daily living and other clinical parameters were tested by Pearson (parametric conditions), Spearman (non-parametric conditions) correlation analysis. To assess the convergent validity of the Londrina ADL Test, we hypothesized that there would be moderate to strong correlations between the Londrina ADL Test and functional capacity, upper extremity muscle strength and functionality, and quality of life. Correlation coefficients were interpreted as strong (> 0.50), moderate (0.30 to 0.50), and weak (0.20 to 0.30). Statistical significance was set at *p* < 0.05. The statistical analyses were conducted using the IBM SPSS software (Version 25.0, IBM Corp., Armonk, USA) [21].

## Results

This study included 39 patients diagnosed with SSc and 30 healthy individuals whose characteristics are described in Table 1. There were no significant differences in age, gender, body mass index, marital status and cigarette consumption between the groups. Patients with SSc, 29 (74.3%) had limited cutaneous systemic sclerosis (lcSSc), 10 (25.6%) had diffuse cutaneous systemic sclerosis (dcSSc). Twenty-four (61.54%) patients had lung involvement and 3 (7.69%) patients had cardiac involvement.The score of the MSS was 5.53 ± 2.78. A significant difference was found between the two groups for the total duration of the Londrina ADL Protocol and the five stations (*p* < 0.001). The median score for the total duration of the Londrina ADL Protocol was 319 (289–367) s in the patient group and 263 (230–301.75) s in the healthy group. The Londrina ADL Protocol parameters of the individuals participating in the study are given in Table 2. In the Londrina ADL Protocol, oxygen saturation analysis was performed with 25 subjects in the study group and 30 subjects in the healthy group. Significant differences were found between the two groups for the change values of systolic blood pressure and dyspnea score (*p* < 0.05). It was observed that the energy expended during the Londrina ADL Protocol was similar in both groups.

**Table 1:** Characteristics of participants

	SSc Group (*n* = 39)	Healthy Group (*n* = 30)	
	X̄ ± S/Median(IQR)	X̄ ± S/Median(IQR)	*p*
Age (years)	47 (39.0–63.0)	46 (38.75–53.25)	0.367^b^
Height (cm)	163.9 ± 9.55	167.83 ± 7.71	0.07^a^
Weight (kg)	67.54 ± 13.77	76.33 ± 14.57	0.013^a^
BMI (kg/m^2^)	23.9 (21.23–28.1)	26.75 (22.4–30.45)	0.193^b^
Occupation *n* (%) Working Not working	14 (35.90) 25 (64.10)	19 (63.33) 11 (36.67)	0.044^c^
Marital Status *n* (%) Married Single	27 (69.23) 12 (30.77)	22 (73.33) 8 (26.67)	0.917^c^
Disease Duration (years)	6 (1.5–13)	-	
MSS	5.53 ± 2.78	-	
Disease Subtype n,% Limited SSc Diffuse SSc	29 (74.36) 10 (25.64)	-	
Lung Involvement n(%) Yes No	24 (61.54) 15 (38.46)	-	
Heart Involvement *n* (%) Yes No	3 (7.69) 36 (92.31)	-	
Medication *n* (%) Does not use Steroid + RF Treatment Immunosuppressive + RF Treatment Plaquenil + RF Treatment RF Treatment Only	2 (5.13) 6 (15.38) 12 (30.77) 8 (20.51) 11 (28.21)	-	
Presence of comorbidity *n* (%) No Hypertension Diabetus Mellitus Other	19 (48.72) 7 (17.95) 1 (2.56) 12 (30.77)	16 (53.33) 3 (10.0) 6 (20.0) 5 (16.67)	0.054^c^

*p* < 0.05, a. Independent samples *t* Test; b. Mann–Whitney *U* Test; c. Chi-Square Test; *n* Number, *BMI* Body mass index; *SSc* Systemic Sclerosis, *RF* Reynaud’s Phenomenon. *Non-parametric data are given as median (IQR)

**Table 2:** Comparison of Londrina ADL Test Performance of the Groups

Londrina ADL Protocol	SSc Group (*n* = 39)	Healthy Group (*n* = 30)	
	Median (IQR)	Median (IQR)	*p*^b^
Desk organization (sec)	42 (38–52)	35 (31–39.25)	< 0.001
Walking with weights (sec)	22 (19.25)	20 (17–21.25)	0.001
In-shelf organization (sec)	79 (69–89)	64.5 (56.5–69.25)	< 0.001
Hanging laundry on a rope (sec)	161 (135–178)	132 (102–158)	0.003
18 m walk (sec)	18 (17–20)	16 (15–17)	< 0.001
Total Duration (sec)	319 (289–367)	263 (230–301.75)	< 0.001

*p* < 0.05, b. Mann–Whitney *U* Test. *Non-parametric data are given as median (IQR)

Walking distance for 6MWT was 485 (444–525) m in the patient group and 562 (488.375–588) m in the healthy group. There was a significant difference between the two groups in the parameters of distance walked, percentage of expected distance, and percentage of distance walked (*p* < 0.05). Vital signs were similar for both groups (*p* > 0.05). Oxygen saturation analyses of the 6MWT were performed with 25 participants in the study group and 30 participants in the healthy group. There was a significant difference between the groups in terms of shoulder, hand and finger grip muscle strength for dominant side (*p* < 0.05). A significant difference was found between the groups for Q-Dash Questionnaire score (*p* < 0.05), 9-Hole Peg Test duration (*p* < 0.05), MAS scores (*p* < 0.05). Except for the emotional role difficulty and mental health parameters of the SF-36 questionnaire (*p* > 0.05), differences were found between the groups for the other 7 parameters (*p* < 0.05), (Table 3). The Londrina ADL Protocol showed high test–retest reliability with the ICC = 0.813), (Table 4). A moderate negative correlation was found between the time to complete the Londrina ADL Protocol and 6MWT (*r* = − 0.542, *p* = 0.001), and a weak negative correlation was found between dominant side hand grip strength (*r* = − 0.321, *p* = 0.046) and MAS (*r* = − 0.353, *p* = 0.027) in individuals with SSc. A moderate positive correlation was found between the Londrina ADL Protocol and the 9-Hole Peg Test for the dominant side (*r* = 0.519, *p* = 0.001). No correlation was found between the Londrina ADL Protocol and MSS (*r* = 0.261, *p* = 0.316) and lung (*r* = 0.165, *p* = 0.316)—heart (*r* = 0.047, *p* = 0.776) involvement (Table 5).

**Table 3:** Comparison of 6MWT, Upper Extremity Muscle Strength and Functionality and Quality of Life of the Groups

	SSc Group (*n* = 39)	Healthy Group (*n* = 30)	
	X̄ ± S/Median(IQR)	X̄ ± S/Median(IQR)	*p*
6MWT Distance (meter)	485 (444–525)	562 (488.375–588)	< 0.001^b^
6MWT Distance (percent)	72.59 ± 10.45	80.32 ± 10.19	0.003^a^
Dominant Shoulder flexor muscle strength (kg)	11.94 ± 3.38	16.53 ± 4.17	< 0.001^a^
Dominant Shoulder abductor muscle strength (kg)	10.85 ± 3.45	14.45 ± 3.41	< 0.001^a^
Dominant Hand grip strength (kg)	28 (24.3–34.6)	36.15 (30.6–43.65)	< 0.001^b^
Q-Dash Score	31.8 (9.09–43.2)	5.67 (0–23.27)	< 0.001^b^
Dominant 9 Hole Peg Test Duration (seconds)	25 (21–29)	18 (17–21)	< 0.001^b^
MAS Score	533.69 ± 57.83	649.7 ± 30.03	< 0.001^a^
SF-36 Physical Function	75 (60–85)	87.5 (80–96.25)	< 0.001^b^
SF-36 Mental Health	60 (44–76)	74 (68–85)	0.007^b^

*p* < 0.05, a. Independent samples *t* Test; b. Mann–Whitney *U* Test; *6MWT*, 6 Minute Walk Test; *MAS*, Milliken ADL Scale; *SF-36*, Short Form-36^*^Non-parametric data are given as median (IQR)

**Table 4:** Test–retest reliability analysis of the Londrina ADL Protocol

	1.Test X̄ ± S	2.Test X̄ ± S	*p*^b^	ICC	%95 CI	*p*^d^
Londrina ADL Protocol	332.10 ± 63.66	290.79 ± 59.47	< 0.001	0.813	0.01–0.019	< 0.001

*p* < 0.05, b. Mann–Whitney *U* Test, d. ICC; *ICC*, Intraclass correlation coefficient; *S*, Standard Deviation; *CI*, Confidence Interval

**Table 5:** Relationship of the Londrina Protocol with relevant clinical parameters

	Londrina ADL	Protocol
	*r*	*p*
6MWT Distance (meter)	** − 0.542**	**< 0.001**
Dominant 9 Hole Peg Test Duration (seconds)	**0.519**	**0.001**
Dominant Shoulder flexor muscle strength (kg)	− 0.186	0.257
Dominant Shoulder abductor muscle strength (kg)	− 0.180	0.272
Dominant Hand grip strength (kg)	** − 0.321**	**0.046**
Q-Dash Score	0.093	0.572
MAS Score	** − 0.353**	**0.027**
MSS Score	0.261	0.136
Lung Involvement	0.165	0.316
Heart Involvement	0.047	0.776
SF-36 Physical Function	− 0.149	0.365
SF-36 Mental Health	0.240	0.141

*6MWT*, 6 Minute Walk Test; *MAS*, Milliken ADL Scale; *MSS*, Medsger Severity Scale; *SF-36*, Short Form-36

## Discussion

Londrina ADL Protocol is a reliable and valid method for assessing the performance of individuals with SSc in activities of daily living. In individuals with SSc, as the time to complete the Londrina Protocol increased, walking distance in the 6MWT, MAS scores and dominant side hand grip strength decreased. As the time to complete the Londrina ADL Protocol increased, the duration of the 9-Hole Peg Test also increased. However, other muscle strength values and quality of life were not found to be associated with the time to complete the Londrina ADL Protocol. To the best of our knowledge, this is the first study to assess ADL in individuals with SSc using the Londrina ADL Protocol. Peripheral muscle strength, functional capacity, upper extremity functionality and quality of life were lower in individuals with SSc compared to the healthy group. The time taken to complete the Londrina ADL Protocol was longer in individuals with SSc compared to the healthy group.

There is a limited number of studies investigating performance-related assessment of ADL impairment in this patient group. Nonato et al. performed GAT in individuals with scleroderma and found that they had longer task completion time than healthy group [22]. Similarly, as a result of the Londrina ADL Protocol in patients with COPD, it was observed that the test duration of the patients was longer than that of healthy individuals [11]. While a significant difference was observed in dyspnea, leg fatigue and oxygen saturation values during the Londrina Protocol in COPD patients, a significant difference was observed in dyspnea and SBP change values in our study [11]. This is thought to be an indication that patients had difficulty in performing activities during the test. Although the test duration was longer in our study, there was no statistically significant difference between the two groups for total energy expended during the test. According to this finding, it is thought that individuals with SSc may have expended more energy than healthy group in a given workload.

Pugned et al. showed that 6MWT is a valid and reliabile test in patients with SSc and walking distance was found to be 457 ± 117 m and walking percentage was found to be 74.7 ± 20% [23]. According to Troosters et al., if the expected value of 6MWT was below 82%, it was accepted that exercise capacity was affected [14]. In this context, it is seen that the functional capacity of individuals with SSc in our study may have been negatively affected due to systemic effects and decreased muscle strength, and the decrease in functional exercise capacity supports the existing information in the literature [22, 24] However, 6MWT results may be affected in these patients due to skin and musculoskeletal system involvement. The results should be more carefully interpreted in both the adult and pediatric groups because similar effects may affect the 6MWT result [25, 26].

Previous studies have shown that hand functions are affected due to negative effects on hand grip strength, finger range of motion and dexterity [27–31]. Mathiowetz et al. showed that the normative values of the 9-Hole Peg Test were 18.8 s for the right hand and 20.4 s for the left hand in men aged 40–49 years and 17.3 s for the right hand and 18.4 s for the left hand in women [18]. Skin, joint and muscle involvement is likely to manifest as muscle weakness and hand dexterity is likely to be affected as a result of reduced hand grip strength. Similar results were found in the study conducted by Dag et al. in a pediatric population and it was reported that hand grip strength and functionality were affected [32].

Varju et al. showed that the Q-Dash Questionnaire is a valid and reliable test in patients with SSc and the questionnaire score was 34.0 (20.4–52.2) [33]. Sezgin et al. reported that activities of daily living were mildly affected in individuals with SSc according to the MAS score [34]. Li et al. showed that the evaluation of quality of life of individuals with SSc with SF-36 was examined and it was found that all 8 sub-parameters of SF-36 were lower in the patient group compared to the healthy group [19].

We found an high test–retest reliability of the Londrina Protocol in SSc patients, which is similar to other studies in healthy adults and patients with COPD [11, 35]. Sant'Anna T et al. reported an ICC value of 0.90, while Paes T et al. reported an ICC value of 0.91. Considering that the Londrina ADL Protocol includes the most commonly used activities in daily life, it will be useful for clinicians to monitor disease progression and treatment.

Sant'Anna T et al. found a negative moderately significant correlation between the Londrina Protocol and the 6MWT and concluded that this is normal considering that the Londrina ADL Protocol represents functional performance. In the GAT applied by Nonato et al. in individuals with SSc, a negative correlation was observed between GAT and hand grip strength and 6MWT. The moderately significant correlation between the Londrina ADL Protocol and the 9-Hole Peg Test suggests that ADL scores may be useful in the follow-up of patients with SSc. Since the content of the MAS and the Londrina ADL Protocol overlap, the weak negative correlation between them may be clinically important.

This study has some limitations. Since the individuals included in the study were not evenly distributed according to disease subtypes and some subtypes had relatively fewer individuals, the level of ADL participation could not be determined specifically for subgroups. At the same time, reaching individuals with relatively good functional levels in our study may have limited the full assessment of the impact of ADL and functional capacity. Therefore, including participants with different disease severities and subtypes for future studies may provide more information. Assessing the response to change over time is indeed a critical aspect for evaluating the validity of any test. However, in this study, we focused on the evaluation of the reliability of the Londrina ADL Protocol Test in a cross-sectional design. Therefore, not evaluating the response to change over time and having a cross-sectional design is one of our limitations. For future studies, we think that studies with long-term and prospective designs will be more reliable in evaluating the responsiveness of the Londrina ADL Test to changes over time.

The results obtained by reapplication of the Londrina ADL Protocol under the same conditions at 1-h intervals showed that the Londrina ADL Protocol is a highly reliable assessment method in scleroderma patients. It is thought to be useful for clinicians and researchers to apply this protocol as an effective and objective method.

## Data Availability

The data that support the findings of this study are available from the corresponding author upon reasonable request.
